# High Prevalence of Multidrug-Resistant Haemolytic *Escherichia coli* in Colombian Pig Farms

**DOI:** 10.3390/antibiotics15010078

**Published:** 2026-01-12

**Authors:** Adriana Pulido-Villamarín, Mattia Pirolo, Iliana C. Chamorro-Tobar, Irina Barrientos-Anzola, Carlos Daza, Raúl A. Poutou-Piñales, Mónica Pérez-Vargas, Luca Guardabassi

**Affiliations:** 1 Unidad de Investigaciones Agropecuarias (UNIDIA), Departamento de Microbiología, Facultad de Ciencias, Pontificia Universidad Javeriana, Bogotá 1117111, Colombia; 2 Center for Research and Technology Transfer of the Pig Sector-Ceniporcino, Asociación Porkcolombia-Fondo Nacional de la Porcicultura, Bogotá 110231, Colombia; ichamorro@porkcolombia.co (I.C.C.-T.); ibarrientos@porkcolombia.co (I.B.-A.); cdaza@porkcolombia.co (C.D.); mperez@porkcolombia.co (M.P.-V.); 3 Facultad de Ciencias Veterinarias, Universidad Nacional del Centro de la Provincia de Buenos Aires (UNICEN), Tandil B7000GHG, Argentina; 4 Department of Veterinary and Animal Sciences, University of Copenhagen, 1870 Frederiksberg, Denmark; mapi@sund.ku.dk (M.P.); lg@sund.ku.dk (L.G.); 5 Grupo de Biotecnología Ambiental e Industrial (GBAI), Departamento de Microbiología, Facultad de Ciencias, Pontificia Universidad Javeriana, Bogotá 111711, Colombia; rpoutou@javeriana.edu.co

**Keywords:** haemolytic *E. coli*, pig production, multidrug resistance, Colombia

## Abstract

**Introduction:** Haemolytic *Escherichia coli* (*E. coli*) is commonly associated with enteric disease in pigs and is frequently used as a phenotypic marker for enterotoxigenic *E. coli* (ETEC). This study aimed to characterise the resistance and virulence profiles of haemolytic *E. coli* isolated from Colombian pig farms. **Methods**: A total of 367 faecal samples from sows and pigs across all production stages were collected and analysed for the presence of haemolytic *E. coli*. Resistance and virulence genes associated with ETEC was detected by multiplex PCR, and antimicrobial susceptibility profiles were determined using broth microdilution and disc diffusion. **Results**: Haemolytic *E. coli* were identified in 40.3% of samples (*n* = 148 non-duplicate isolates), with the highest prevalence observed in growing piglets (47.1%). ETEC occurred in 5.4% of isolates. All isolates exhibited resistance to at least three antimicrobial classes (MDR), with high levels of resistance to tetracycline (98.0%), neomycin (97.3%), chloramphenicol (95.9%), sulfamethoxazole (93.9%), trimethoprim (91.9%), ampicillin (91.9%), nalidixic acid (82.4%), and ciprofloxacin (79.7%). Colistin resistance was observed in 5.4% of isolates, mediated by *mcr*1 or *mcr*3, while cefotaxime resistance (8.8%) was extensively associated with blaCTX-M. **Conclusions**: These findings reveal a concerning burden of MDR *E. coli* in Colombia’s pig-producing regions and indicate that haemolysis alone is a poor indicator of ETEC. Integrating farm-level antimicrobial use data with genomic analyses will be essential to identify drivers of AMR and guide effective stewardship in the Colombian pig industry.

## 1. Introduction

*Escherichia coli* (*E. coli*) is a commensal organism of the intestinal microbiota of animals, but also includes pathogenic variants responsible for important veterinary diseases such as neonatal and post-weaning diarrhoea in pigs [1]. Enterotoxigenic *E. coli* (ETEC) strains are often implicated in these enteric diseases [2], which cause significant economic losses in pig production due to high morbidity and mortality rates, as well as production performance [3,4]. ETEC strains possess a variable repertoire of virulence factors, including fimbriae, mainly F4 and F18, which promote adhesion to the intestinal mucosa. In addition, they produce one or more toxins, such as heat-stable toxins (STxa, STxb) and heat-labile toxin (LT) [4,5,6], which induce secretory diarrhoea by altering water and electrolyte balance in the small intestine. ETEC strains usually exhibit haemolytic activity, and haemolysis on blood agar is used as a practical phenotypic marker in diagnostic microbiology and epidemiological studies [7].

Antimicrobial therapy for pig enteric infections has become increasingly challenging due to the recent emergence of clinical *E. coli* strains resistant to neomycin, an aminoglycoside commonly used for treatment [8]. Although colistin was previously used for this indication, its widespread resistance along the food chain and association with multidrug-resistant (MDR) infections in humans have led countries worldwide to introduce bans or restrictions on its use in food-producing animals [9,10]. In Colombia [11] and other Latin American countries [9,10], the use of colistin as a growth promoter has been prohibited in line with global efforts to regulate the use of critically important antimicrobials in livestock production [12]. However, data on antimicrobial resistance (AMR) in porcine *E. coli* remain limited, hindering efforts to characterize local resistance trends and to develop evidence-based antimicrobial stewardship strategies.

Colombia ranks fourth among Latin American pork-producing countries, with pig production contributing approximately 6% the country’s agricultural gross domestic product [13]. Before this study, only one investigation had examined the AMR and virulence traits of haemolytic *E. coli* isolates from diarrhoeic pigs in Colombia, particularly in the Valle del Cauca region. Haemolytic isolates displayed combinations of adhesion factors F6–F18 and F6–F41, in the absence of detectable toxin production, along with resistance to several antimicrobial classes, including aminoglycosides, fluoroquinolones, and cephalosporins [14]. Based on these limited data and in the updated list of priority pathogens AMR for the World Health Organisation (WHO), in which Enterobacteriaceae microorganisms, such as *E. coli*, are classified as critical and high priority [15], the present study aimed to characterise the AMR and virulence profiles of haemolytic *E. coli* isolated from Colombian pig farms in Antioquia, Cundinamarca, Valle del Cauca, and Meta. These regions were selected based on their high production volumes and strategic importance to the national sector.

## 2. Results

### 2.1. Haemolytic E. coli Prevalence

A total of 367 faecal samples were collected from pigs of different age groups across nine farms, including 175 in Antioquia (47.7%), 81 in Cundinamarca (22.1%), 60 in Meta (16.3%), and 51 in Valle del Cauca (13.9%), resulting in the isolation of 148 haemolytic *E. coli* from 40.3% of the samples. When analysed by region, haemolytic *E. coli* occurred in 48.0% of samples from Antioquia, 41.2% from Valle del Cauca, 30.9% from Cundinamarca, and 30.0% from Meta. Haemolytic strains were most prevalent among growing piglets, followed by suckling piglets, finishing pigs, and sows (Table 1).

### 2.2. Genetic Characterization

Screening for the main ETEC fimbriae (F4 and F18) and toxins (STxa, STxb, and LTx) revealed that only eight isolates (5.4%) were ETEC, including five F4-, Sta- and STb-positive strains, and three F18- and Sta-positive ones. The original gel electrophoresis appears in Figure S1.

These were detected exclusively in growing piglets from four farms in two departments, namely Antioquia and Valle del Cauca. Virulence factors were undetected in isolates from sows, suckling piglets, and finishing pigs from Cundinamarca and Meta.

**Figure 1:**
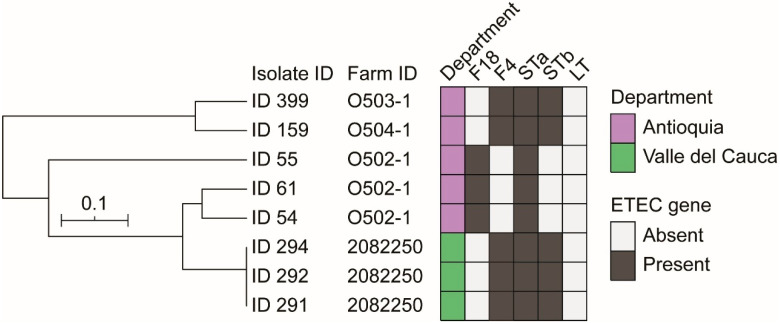
MLVA fingerprint analysis of eight ETEC isolates from pre-weaning piglet samples. The phylogeny was generated with GelJ v2 using UPGMA and the Dice coefficient. The original gel electrophoresis is provided in Figure S2.

Multiple Locus Variable Analysis (MLVA) profiling of the ETEC isolates revealed a close relationship between isolates from the same farm (ID O502-1 and ID 2082250) or between different farms in the same Department (ID O503-1 and ID O504-1) (Figure 1).

### 2.3. Antimicrobial Resistance Profiling

Following the Clinical and Laboratory Standards Institute [16], susceptibility results by broth microdilution revealed that all isolates were MDR [95% CI, 97.5–100.0%], defined as by resistance to at least one agent in three or more antimicrobial categories [17], with high levels of resistance recorded for tetracycline (TET; 98.0%), chloramphenicol (CHL, 95.9%), sulfamethoxazole (SMX, 93.9%), trimethoprim (TMP, 91.9%), ampicillin (AMP, 91.9%), nalidixic acid (NAL, 82.4%), and ciprofloxacin (CIP, 79.7%). Resistance to gentamicin (GEN), azithromycin (AZI), tigecycline (TGC), cefotaxime (CTX), colistin (COL) and meropenem (MEM) was observed in 38.5%, 15.5%, 15.5%, 8.8%, 5.4%, and 0.7% of isolates, respectively. All isolates were susceptible to amikacin (AMI) and piperacillin/tazobactam (TAZ). A total of 48 different resistance profiles were found; the six most common ones (those with >2% of isolates) are reported in Table 2.

To extend the antimicrobial coverage of the susceptibility assessment, eight additional antimicrobial drugs were tested by disk diffusion. Resistance to neomycin (NEO), sulfamethoxazole (STX), amoxicillin + clavulanic acid (AMC), cefepime (FEP), and cefoxitin (FOX) was observed in 97.3%, 89.9% 7.4%, 3.4%, 3.4% and 0.7% of isolates, respectively, while no resistance to ertapenem (ETP) and MEM was detected.

Isolates showing resistance to COL (*n* = 8) and CTX (*n* = 13) were screened for the most common COL resistance and ESBL-encoding genes, respectively. Four of the eight COL-resistant isolates carried *mcr3*, two *mcr1*, and the remaining two were negative for all the *mcr* genes screened (*mcr1* to *mcr5*). Of the 13 CTX-resistant isolates, 12 carried *bla_CTX-M_*, two of which, in combination with *bla_CMY-2_*, while one isolate carried *bla_CMY-2_* alone.

Both colistin and cefotaxime resistance, as well as the respective resistance genes, were present throughout the production stages analysed in the departments of Antioquia, Cundinamarca, and Valle del Cauca. None of the isolates obtained in Meta exhibited these last characteristics.

## 3. Discussion

This study provides the first comprehensive evidence of AMR and virulence traits in haemolytic *E. coli* strains from all stages of pig production in the crucial producing regions of Colombia. Although haemolysis has traditionally been considered a marker of ETEC [1], our results indicate that this phenotypic trait alone is not a reliable predictor. Only a minority of haemolytic isolates carried ETEC-associated virulence factors, suggesting that haemolytic *E. coli* populations in Colombian herds are extensively composed of commensal or other pathogenic variants rather than classic ETEC pathotypes. This observation aligns with findings from other studies reporting that haemolytic activity can also occur in non-ETEC strains lacking key virulence determinants, such as fimbriae and enterotoxins [18]. Schierack et al. (2011) [18] demonstrated that a large proportion of haemolytic isolates from both healthy and diarrhoeic pigs in Germany lacked typical intestinal virulence-associated genes but frequently carried traits characteristic of extraintestinal pathogenic *E. coli* (ExPEC). Future genomic studies will be essential to determine whether the Colombian isolates share genetic backgrounds with known ExPEC lineages and to clarify their potential role as reservoirs of AMR within pig production systems, as well as their possible zoonotic potential. 

To date, there are few reports related to ETEC strains in pig production in Colombia. In 2023, Pabón-Rodríguez et al. (2023) [14] reported an *E. coli* prevalence of 67.5% (52/77) and a β-haemolytic capacity of 11.5% (6/52) in farms in the Valle del Cauca region, based on samples from diarrhoeic suckling or pre-weaned piglets. Some of these strains carried fimbriae F6 (4/6), F18 (2/6), and F41 (2/6), and none of the isolates presented genes encoding toxins [19,20]. Because their sampling targeted diseased piglets rather than apparently healthy animals, direct comparison of prevalence values is not appropriate; however, the higher detection rate in that study is consistent with the expectation that haemolytic strains with pathogenic potential are more frequent in diarrhoeic pigs. In addition, methodological differences between the two studies, particularly in the virulence gene panels used for molecular screening, may also account for variations in the detection of ETEC-associated markers. Here, the prevalence of β-haemolytic *E. coli* in the same region was 41.2%, with three isolates (5.9%) being ETEC (F18-, STxa-, and STxb-positive). The virulent types detected in this study are similar to those reported by De Lorenzo et al. (2018) in Brazil, where F18-STxa-STxb and F18-STxa patterns were detected in 5.7% and 7.5% of cases, respectively [3]. In Spain, the most prevalent types were STEC and ETEC with STa-STb-Stx2e-F18 (86.7%); Stx2e-F18 (70%); LT-STb-F4 (37.3%); and LT-STa-STb-F18 (18.6%) patterns [21], contrasting with the findings of the present study, as the LT was undetected in any of the isolates.

Susceptibility testing revealed an MDR phenotype in all isolates, with high resistance rates > 90% in TET, NEO, SMX, TMP and AMP. Our findings are consistent with previous reports from Antioquia [22], which may reflect historical selection pressure exerted by the frequent and prolonged use of these antimicrobials in swine production. In addition, resistance to fluoroquinolones was detected in approximately 80% of the isolates, representing a significant public health concern given the critical importance of this class in human medicine [12]. The detection of third-generation cephalosporin resistance in approximately 9% of isolates, associated with extended-spectrum β-lactamases (ESBLs) of the CTX-M type, is equally alarming. These enzymes are among the most widespread and clinically significant ESBLs in human medicine, where they are associated with treatment failures and increased morbidity and mortality [23]. Both fluoroquinolones and third-generation cephalosporins are high-priority critically important antimicrobials according to the World Health Organization [12], based on the AMEG classification [24], their employment must be restricted in food-producing animals to minimise the risk of selecting and disseminating AMR to humans. The coexistence of fluoroquinolone and ESBL-mediated resistance to third and fourth-generation cephalosporins (8/13 as our case) further underscores the potential for co-selection and horizontal dissemination of AMR phenotypes and determinants of high medical importance within pig production systems. These findings are of particular concern, as pigs can act as reservoirs of resistant bacteria and mobile resistance genes that may be transmitted to humans through direct animal contact, environmental contamination, or the food chain.

The presence of genes *bla_CTX-M_* and *bla_CMY-2_* in our isolates contrasts with Brazil, where *bla_SHV_*, *bla_GES_*, *bla_VEB_*, and *bla_CTX-M-Gp1_* were reported, not only in pig faeces, but also in soil and water of swine farms, indicating the spread of *E. coli* resistant to third-generation cephalosporins within and beyond production facilities [25]. Despite its restriction, COL is still used to treat gastrointestinal diseases in veterinary settings [4,26]. In this study, 5.4% of isolates were resistant to this antimicrobial, primarily due to the carriage of the *mcr*3 gene, which differs from previous reports from pig farms where *mcr*1 was the predominant variant [4,19,21,27]. In Colombia, gene *mcr*1 was reported in *E. coli* and *Salmonella enterica* from pigs in an abattoir at the department of Antioquia [28]; and *bla_TEM1-B_* and *bla_CMY-2_* were found in *S. typhimurium* isolated from water and faeces of pigs at swine farms [29]. Together, these findings underscore the need for AMR surveillance within a “One Health” framework, since the dissemination of MDR strains carrying these AMR determinants poses a potential zoonotic risk to public health.

While Table 2 identifies the antimicrobial classes to which porcine haemolytic *E. coli* most frequently exhibited resistance, these phenotypic data alone do not allow inferences about actual antimicrobial use or selective pressures within production systems. To understand AMR patterns, granular antimicrobial use data are required for pig production under local conditions, ideally disaggregated to the farm or production-unit level rather than limited to national or regional aggregates. AMU can vary substantially with production practices, genetics, veterinary oversight, and management protocols; consequently, farm-level metrics (e.g., mg/PCU, DDDvet/DCDvet per class and indication) would enable more precise linkage between exposure and resistance outcomes; this is a key limitation of the present study, which precludes direct attribution of the observed AMR patterns to specific antimicrobial use practices.

## 4. Materials and Methods

### 4.1. Study Design, Farm Selection, and Sample Collection

A cross-sectional study employing simple random sampling involved 9 commercial farms (≥100 sows and ≥600 fattening pigs), located in several Colombian Departments: Antioquia (*n* = 4), Cundinamarca (*n* = 3), Valle del Cauca (*n* = 1), and Meta (*n* = 1). In Colombia, the classification of farms (as those commercial farms used for this study) depends on biosecurity levels, production scale, animal health status, health measures, and management practices, in full compliance with the guidelines established by the Colombian Agricultural Institute (ICA) for all legal pig farms in the country [30]. Sampling focused on all stages of the production cycle, including lactating sows, suckling piglets, and fattening (growing and finishing pigs). The number of animals per pen ranged from 10 to 20, depending on the production stage. Fresh faecal samples were collected from the ground in the pen and from the middle of at least five fresh droppings, avoiding the surface layer, potentially contaminated by flies, and the lower layer in direct contact with the ground. Individual faecal samples were collected from sows to ensure a detailed assessment of this specific population. The material was placed in an airtight sealed bag, maintained refrigerated in a container, and transported to the laboratory within 4 to 6 h.

### 4.2. Bacteria Identification and Molecular Characterization

Faecal samples were homogenised and cultured on MacConkey agar and blood agar (Scharlau; Scharlab S.L., Sentmenat, Spain) with 5% sheep blood to evaluate haemolytic activity typical of ETEC. After 24 h of incubation at 37 °C, haemolytic colonies were sub-cultured on MacConkey agar to confirm their typical *E. coli* morphology (dry, pink, lactose-positive colonies) and to ensure culture purity before further characterisation, and to identify to the species level through MAL-DI-TOF MS mass spectrometry (Vitek^®^MSTM, bioMérieux, Montreal, QC, Canada).

The boiling method enables the extraction of *E. coli* isolates DNA [31,32]. ETEC screening was performed by PCR targeting the main fimbria types (F4 and F18) and toxins (STxa, STxb, and LTx), as previously described (Table S1) [33,34]. Confirmed ETEC strains were profiled by Multiple Locus Variable Analysis (MLVA) [31] (Table S2). MLVA profiles were analysed using GelJ v2 [35], and the phylogenetic tree was constructed after utilising the unweighted pair group method with arithmetic averages (UPGMA) after clustering with Dice similarity index (3% tolerance). Phylogeny was visualized with iTOL v6 [36].

### 4.3. Antimicrobial Susceptibility Testing

Minimum inhibitory concentration (MIC) was determined by broth microdilution in Mueller-Hinton broth using the Sensititre™ automated system, following the manufacturer’s instructions. Microdilution plates for Gram-negative bacteria (EUVSEC3, Thermo Scientific™, Waltham, MA, USA). The panel included 15 antimicrobials, including amikacin (AMK), ampicillin (AMP), azithromycin (AZ), cefotaxime (CTX), ceftazidime (CAZ), chloramphenicol (CHL), ciprofloxacin (CIP), colistin (COL), gentamicin (GEN), meropenem (MER), nalidixic acid (NAL), sulfamethoxazole (SMX), tetracycline (TET), tigecycline (TGC), and trimethoprim (TMP). After incubation at 37 °C ± 2 for 18–20 h, plate readings were performed using Vision™ equipment (Tokyo, Japan), and the susceptibility was evaluated with Sensititre™ SWIN™ v2.4 software.

To assess susceptibility to a wide range of antimicrobials, we performed complementary disk diffusion testing (Oxoid, Hampshire, UK) using eight additional agents of relevance in human or veterinary medicine: meropenem (MEM; 10 mg), ertapenem (ETP; 10 mg), imipenem (IMP; 10 mg), cefoxitin (FOX; 30 mg), cefepime (FEP; 30 mg), neomycin (NEO; 10 mg), trimethoprim-sulfamethoxazole (STX; 25 mg), amoxicillin + clavulanic acid (AMC; 30 mg).

MICs and inhibition zone diameters were interpreted according to CLSI standards [16]. Strains classified as intermediate resistant (now renamed ‘susceptible, increased exposure’) were included in the same group as susceptible when reporting percentages of resistant strains. In addition, strains showing resistance to at least one agent from three or more antimicrobial classes were classified as MDR [17].

### 4.4. AMR Genes Screening

Colistin-resistant isolates were screened for colistin resistance genes (*mcr*-1 to *mcr*-5) by PCR following the protocol described by Rebelo et al. (2018) (Table S3) [37]. Third-generation cephalosporin-resistant isolates were screened for ESBL genes by PCR as previously described (Table S4) [37], including testing for *bla_CTX-M_*, *bla_SHV_*, *bla_TEM_*, *bla_CMY-1_*, *bla_CMY-2_*, *bla_OXA-1_*, and *bla_OXA-2_*.

### 4.5. Data Analysis

Data management and statistical calculations were performed using Microsoft Excel and R version 4.3.1. Prevalence and frequency of haemolytic *E. coli* and AMR profiles were calculated as mean values of positive samples over the total number of samples for each group, with 95% confidence intervals (95% CI) determined using the Wilson Score method.

## 5. Conclusions

ETEC-associated virulence factors were only detected in 5.4% of haemolytic *E. coli isolates* from Colombian pig herds, demonstrating that haemolysis alone is an unreliable marker of ETEC. All haemolytic isolates exhibited an MDR phenotype, indicating widespread resistance to critically important antimicrobials such as fluoroquinolones, third-generation cephalosporins and colistin. Future research should integrate detailed farm-level antimicrobial use data with genomic analyses to enable the identification of actionable drivers of AMR and support the development of targeted stewardship strategies and regulatory frameworks to mitigate AMR in Colombian pig production.

## Figures and Tables

**Table 1:** Prevalence (%) of haemolytic *E. coli* by age group in four departments of Colombia.

Department	Sow	Suckling Piglets	Growing Piglets	Finishing Pigs
	% (Proportion)			
Cundinamarca	25 (4/16)	58.3 (7/12)	27.3 (6/22)	25.8 (8/31)
Antioquia	34.5 (10/29)	34.5 (10/29)	59.4 (38/64)	49.1 (26/53)
Meta	10 (1/10)	40 (4/10)	30 (6/20)	35 (7/20)
Valle del Cauca	50 (4/8)	37.5 (3/8)	46.7 (7/15)	35 (7/20)
Total	30.2% (19/63)	40.7% (24/59)	47.1% (57/121)	38.7% (48/124)
CI 95% ^a^	[20.2–42.4]	[29.1–53.4]	[38.4–56.0]	[30.6–47.5]

^a^ 95% confidence interval (CI).

**Table 2:** Six main antimicrobial resistance profiles observed in haemolytic *E. coli* (*n* = 148).

Resistance Profile	No. of Isolates (%)
AMP-CHL-CIP-NAL-SMX-TET-TMP	36 (24.3)
AMP-CHL-CIP-GEN-NAL-SMX-TET-TMP	31 (20.9)
AMP-CHL-SMX-TET-TMP	7 (4.7)
AMP-AZI-CHL-CIP-GEN-NAL-SMX-TET-TMP	6 (4.1)
AMP-CHL-CIP-NAL-SMX-TET-TGC-TMP	5 (3.4)
AMP-AZI-CHL-CIP-NAL-SMX-TET-TMP	4 (2.7)

**Abbreviation**: AMP, Ampicillin; AZI, Azithromycin; CHL, Chloramphenicol; CIP, Ciprofloxacin; GEN, Gentamicin; NAL, Nalidixic Acid; SMX, Sulfamethoxazole; TET, Tetracycline; TGC, Tigecycline; TMP, Trimethoprim.

## Data Availability

Research data are available on request.

## Supplementary Materials

The following supporting information can be downloaded at: https://www.mdpi.com/article/10.3390/antibiotics15010078/s1. Refs. [31,33,34,37,38] are cited in the Supplementary Materials.

## References

[1] Schierack P., Steinrück H., Kleta S., Vahjen W. (2006). Virulence factor gene profiles of *Escherichia coli* isolates from clinically healthy pigs. Appl. Environ. Microbiol..

[2] Fratamico P.N., DebRoy C., Liu Y., Needleman D.S., Baranzoni G.M., Feng P. (2016). Advances in molecular serotyping and subtyping of *Escherichia coli*. Front. Microbiol..

[3] De Lorenzo C., de Andrade C.P., Machado V.S.L., Bianchi M.V., Rolim V.M., Cruz R.A.S., Driemeier D. (2018). Piglet colibacillosis diagnosis based on multiplex polymerase chain reaction and immunohistochemistry of paraffin-embedded tissues. J. Vet. Sci..

[4] Göpel L., Prenger-Berninghoff E., Wolf S.A., Semmler T., Bauerfeind R., Ewers C. (2023). Occurrence of mobile colistin resistance genes *mcr*-1–*mcr*-10 including novel mcr gene variants in different pathotypes of porcine *Escherichia coli* isolates collected in Germany from 2000 to 2021. Appl. Microbiol..

[5] Francis D.H. (2002). Enterotoxigenic *Escherichia coli* infection in pigs and its diagnosis. J. Swine Health Prod..

[6] Zhang W., Zhao M., Ruesch L., Omot A., Francis D. (2007). Prevalence of virulence genes in *Escherichia coli* strains recently isolated from young pigs with diarrhea in the US. Vet. Microbiol..

[7] Frydendahl K. (2002). Prevalence of serogroups and virulence genes in *Escherichia coli* associated with postweaning diarrhoea and edema disease in pigs and a comparison of diagnostic approaches. Vet. Microbiol..

[8] Subramani P., Menichincheri G., Pirolo M., Arcari G., Kudirkiene E., Polani R., Carattoli A., Damborg P., Guardabassi L. (2023). Genetic background of neomycin resistance in clinical *Escherichia coli* isolated from Danish pig farms. Appl. Environ. Microbiol..

[9] Ahmed M.O., Abouzeed Y.M., Daw M.A. (2025). Global initiatives to phase-out colistin use in food-producing animals. Open Vet. J..

[10] Da Silva R.A., Arenas N.E., Luiza V.L., Bermudez J.A.Z., Clarke S.E. (2023). Regulations on the use of antibiotics in livestock production in South America: A comparative literature analysis. Antibiotics.

[11] Instituto Colombiano Agropecuario—I.C.A (2024). Resolución 2024R00010003, Por la cual se Prohíbe en el Territorio Nacional la Importación, Fabricación, Registro, Comercialización y Uso de Polimixina E (colistina) y Polimixina B en Cualquiera de sus Formas Químicas en Especies Animales. https://www.ica.gov.co/normatividad/normas-ica/resoluciones-oficinas-nacionales/2024/2024r00010003.

[12] World Health Organization (2024). WHO List of Medically Important Antimicrobials. A Risk Management Tool for Mitigating Antimicrobial Resistance Due to Non-Human Use.

[13] Castro C.A. (2025). Analysis of Latin American Swine Farming Performance in 2024 and Outlook for 2025. https://www.pig333.com/articles/analysis-of-latin-american-swine-farming-performance-2024-2025_21332/.

[14] Pabon-Rodriguez O.V., Lopez-Lopez K., Casas-Bedoya G.A., Mogollon-Galvis J.D., Serna-Cock L. (2023). Adhesion factors and antimicrobial resistance of *Escherichia coli* strains associated with colibacillosis in piglets in Colombia. Vet. World.

[15] Sati H., Carrara E., Savoldi A., Hansen P., Garlasco J., Campagnaro E., Boccia S., Castillo-Polo J.A., Magrini E., Garcia-Vello P. (2025). The WHO Bacterial Priority Pathogens List 2024: A prioritisation study to guide research, development, and public health strategies against antimicrobial resistance. Lancet Infect. Dis..

[16] (2018). Performance Standards for Antimicrobial Disk and Dilution Susceptibility Tests for Bacteria Isolated from Animals.

[17] Magiorakos A.P., Srinivasan A., Carey R.B., Carmeli Y., Falagas M.E., Giske C.G., Harbarth S., Hindler J.F., Kahlmeter G., Olsson-Liljequist B. (2012). Multidrug-resistant, extensively drug-resistant and pandrug-resistant bacteria: An international expert proposal for interim standard definitions for acquired resistance. Clin. Microbiol. Infect..

[18] Schierack P., Weinreich J., Ewers C., Tachu B., Nicholson B., Barth S. (2011). Hemolytic porcine intestinal *Escherichia coli* without virulence-associated genes typical of intestinal pathogenic *E. coli*. Appl. Environ. Microbiol..

[19] Luo Q., Wang Y., Xiao Y. (2020). Prevalence and transmission of mobilized colistin resistance (*mcr*) gene in bacteria common to animals and humans. Biosaf. Health.

[20] Chandler J.C., Franklin A.B., Bevins S.N., Bentler K.T., Bonnedahl J., Ahlstrom C.A., Bisha B., Shriner S.A. (2020). Validation of a screening method for the detection of colistin-resistant *E. coli* containing *mcr*-1 in feral swine feces. J. Microbiol. Methods.

[21] García-Meniño I., Mora Gutiérrez A. (2021). Clones de *E. coli* implicados en colibacilosis porcina en España y antibiorresistencias. SUIS.

[22] Mantilla J.F., Villar D., Gómez-Beltrán D.A., Vidal J.L., Chaparro-Gutiérrez J.J. (2021). High antimicrobial resistance in *Salmonella* spp. and *Escherichia coli* isolates from swine fecal samples submitted to a veterinary diagnostic laboratory in Colombia. Rev. Colomb. Cienc. Pecu..

[23] Pitout J.D., Nordmann P., Laupland K.B., Poirel L. (2005). Emergence of Enterobacteriaceae producing extended-spectrum beta-lactamases (ESBLs) in the community. J. Antimicrob. Chemother..

[24] European Medicines Agency (EMA), Committee for Medicinal Products for Veterinary Use (CVMP), Committee for Medicinal Products for Human Use (CHMP) (2025). Categorisation of Antibiotics in the European Union.

[25] Rueda Furlan J.P., Guedes Stehling E. (2018). Detection of beta-lactamase encoding genes in feces, soil and water from a Brazilian pig farm. Environ. Monit. Assess..

[26] Lekagul A., Tangcharoensathien V., Yeung S. (2019). Patterns of antibiotic use in global pig production: A systematic review. Vet. Anim. Sci..

[27] Brisola M.C., Crecencio R.B., Bitner D.S., Frigo A., Rampazzo L., Stefani L.M., Faria G.A. (2019). *Escherichia coli* used as a biomarker of antimicrobial resistance in pig farms of Southern Brazil. Sci. Total Environ..

[28] Palacio-Arias C.A., Cienfuegos-Gallet A.V., Fernández-Silva J.A., Vásquez-Jaramillo L. (2023). *Escherichia coli* y *Salmonella* spp. portadoras de *mcr*-1 en planta de beneficio porcino, Medellín (Colombia). Rev. MVZ Córdoba.

[29] Chamorro-Tobar I.C., Pulido-Villamarín A., Carrascal-Camacho A.K., Barrientos-Anzola I., Wiesner M., Hernández-Toro I., Alban L., Olsen J.E., Dalsgaard A., Hounmanou Y.M.G. (2024). Phenotypic and genotypic characterization of antimicrobial resistance in *Salmonella enterica* Serovars from colombian pig farms. Appl. Microbiol..

[30] Instituto Colombiano Agropecuario (ICA) (2022). Censo Nacional Porcino por Departamento Según Categoría. https://www.ica.gov.co/areas/pecuaria/servicios/epidemiologia-veterinaria/censos-2016/censo-2018/censo-porcinos-2022.aspx.

[31] Caméléna F., Birgy A., Smail Y., Courroux C., Mariani-Kurkdjian P., Le Hello S., Bonacorsi S., Bideta P. (2019). Rapid and simple universal *Escherichia coli* genotyping method based on Multiple-Locus Variable-Number Tandem- repeat analysis using single-tube multiplex PCR and standard gel electrophoresis. Appl. Environ. Microbiol..

[32] Subramani P., Pirolo M., Haugegaard S., Skarbye A.P., Conrady B., Pedersen K.S., Guardabassi L., Damborg P. (2023). Neomycin resistance in clinical *Escherichia coli* from Danish weaner pigs is associated with recent neomycin use and presence of F4 or F18 fimbriaes. Prev. Vet. Med..

[33] Do K.H., Byun J.W., Lee W.K. (2020). Virulence genes and antimicrobial resistance of pathogenic *Escherichia coli* isolated from diarrheic weaned piglets in Korea. J. Anim. Sci. Technol..

[34] Zajacova Z.S., Konstantinova L., Alexa P. (2012). Detection of virulence factors of *Escherichia coli* focused on prevalence of EAST1 toxin in stool of diarrheic and non-diarrheic piglets and presence of adhesion involving virulence factors in astA positive strains. Vet. Microbiol..

[35] Heras J., Dominguez C., Mata E., Pascual V., Lozano C., Torres C., Zarazaga M. (2015). GelJ—A tool for analyzing DNA fingerprint gel images. BMC Bioinform..

[36] Letunic I., Bork P. (2024). Interactive Tree of Life (iTOL) v6: Recent updates to the phylogenetic tree display and annotation tool. Nucleic Acids Res..

[37] Rebelo A.R., Bortolaia V., Kjeldgaard J.S., Pedersen S.K., Leekitcharoenphon P., Hansen I.M., Guerra B., Malorny B., Borowiak M., Hammerl J.A. (2018). Multiplex PCR for detection of plasmid-mediated colistin resistance determinants, *mcr*-1, *mcr*-2, *mcr*-3, *mcr*-4 and mcr-5 for surveillance purposes. Eurosurveillance.

[38] Hasman H., Mevius D., Veldman K., Olesen I., Aarestrup F.M. (2005). beta-Lactamases among extended-spectrum beta-lactamase (ESBL)-resistant *Salmonella* from poultry, poultry products and human patients in The Netherlands. J. Antimicrob. Chemother..

