# Supplementary material for: High Prevalence of Multidrug-Resistant Haemolytic Escherichia coli in Colombian Pig Farms
Source: Antibiotics (Basel). 2026 Jan 12;15(1):78. doi: 10.3390/antibiotics15010078 (PMC12837919; doi:10.3390/antibiotics15010078)
Supplement: Supplementary file 1 [file antibiotics-15-00078-s001.zip › antibiotics-4039695-supplementary.pdf]

SUPLEMENTARY INFORMATION

Table S1. Primers used for multiplex PCR for the detection of virulence factors [33, 34].

Primer	Sequence (5'-3')	Fragment size (bp)
F4-F	TGAATGACCTGACCAATGGTGGAACC	484
F4-R	GCGTTTACTCTTTGAATCTGTCCGAG	
F18-F	TGGCACTGTAGGAGATACCATTCAGC	334
F18- R	GGTTTGACCACCTTTCAGTTGAGCAG	
LT-F	ACGGCGTTACTATCCTGTCTATGTGC	275
LT-R	TTGGTCTCGGTCAGATATGTGATTCT	
STa-F	GTCAGTCAACTGAATCACTTGACTCT	152
STa-R	CATGGAGCACAGGCAGGATTACAACA	
STb-F	GCTACAAATGCCTATGCATCTACACA	125
STb-R	CATGCTCCAGCAGTACCATCTCTAAC	

The strains from the University of Copenhagen were used as positive controls: JEO-5640: positive for F4, LT, STa, STb; and F.1.7.73: positive for F18, LT, STb.

Running conditions were: 1 cycle of denaturation at 95 °C for 15 min, followed by 25 cycles of denaturation at 94 °C for 30 sec, annealing at 63 °C for 30 sec, and elongation at 72 °C for 90 sec, and a final cycle of elongation at 72 °C for 10 min.

Table S2. Primers used for multiplex MLVA [31].

PCR	Primer	Sequence (5'-3')	Fragment size (bp)
ECMLV	ECMLV1-F	TCCCTGGACAAACCAGGACTG	162–1,597
	ECMLV1-R1	CGTGCGGACTTATGAGAAAG	
	ECMLV1-R2	CGTGCGGGCTTATGAAAAAG	
ECMLV2	ECMLV2-F	GAAACAGGCCCAGGCTACAC	575–869
	ECMLV2-R	CTGGCGCTGGTTATGGGTAT	
ECMLV3	ECMLV3-F	TTCAGGAAATGGATAAAGTAGT	616–1,157
	ECMLV3-R	GGGAGTATGCGGTCAAAAGC	
ECMLV4	ECMLV4-F	ACAACCGGCTGGGGCGAATCC	413–539
	ECMLV4-R	GTCAGCAAATCCAGAGAAGGCA	
ECMLV5	ECMLV5-F	GCGGCGCTGAAGAAGAAAGC	375–438
	ECMLV5-R	CTCCCGGCAGGCGAAGCATTGT	
ECMLV6	ECMLV6-F	CAAAGAGCAATAACACTTTTAGCA	102–149
	ECMLV6-R	GCAGCAGGGACAACGGAAGCTAA	
ECMLV7	ECMLV7-F	GTGAAGGATAAGCTGCATTTGTCA	176–211
	ECMLV7-R	GCCTGACGCTAAAGATAAAGAAGA	

Running conditions were: 1 cycle of denaturation at 95 °C for 15 min, followed by 25 cycles of denaturation at 94°C for 30 sec, annealing at 59 °C for 90 sec, and elongation at 72 °C for 90 sec, and a final cycle of elongation at 72 °C for 10 min.

Table S3. Primers used for multiplex PCR for detection of *mcr*-1, *mcr*-2, *mcr*-3, *mcr*-4 and *mcr*-5 genes [37].

Primer	Sequence (5'-3')	Target gene	Fragment size (bp)
mcr1_320bp_fw<br>mcr1_320bp_rev	AGTCCGTTTGTTCTTGTGGC<br>AGATCCTTGGTCTCGGCTTG	*mcr*-1	320
mcr2_700bp_fw<br>mcr2_700bp_rev	CAAGTGTGTTGGTCGCAGTT<br>TCTAGCCCGACAAGCATACC	*mcr*-2	715
mcr3_900bp_fw<br>mcr3_900bp_rev	AAATAAAAATTGTTCCGCTTATG<br>AATGGAGATCCCCGTTTTT	*mcr*-3	929
mcr4_1100bp_fw<br>mcr4_1100bp_rev	TCACTTTCATCACTGCGTTG<br>TTGGTCCATGACTACCAATG	*mcr*-4	1,116
MCR5_fw<br>MCR5_rev	ATGCGGTTGTCTGCATTTATC<br>TCATTGTGGTTGTCCTTTTCTG	*mcr*-5	1,644

Running conditions were: 1 cycle of denaturation at 95 °C for 5 min, followed by 25 cycles of denaturation at 95°C for 30 sec, annealing at 58 °C for 90 sec, and elongation at 72 °C for 60 sec, and a final cycle of elongation at 72 °C for 10 min.

Table S4. Primers used for multiplex PCR for BLEE genes [38].

Primer	Sequence (5'-3')	Target gene	Fragment size (bp)
757<br>821	GCGGAACCCCTATTTG<br>TCTAAAGTATATATGAGTAAACTTGGTCTGAC	*bla*TEM	964
1113<br>796	GTGAATACAGAGCCAGACGC<br>GTTGTTTCCGGGTGATGC	*Pamp*C	343
1436<br>1437	TTCGCCTGTGTATTATCTCCCTG<br>TTAGCGTTGCCAGTGYTCG	*bla*SHV	854
1354<br>1355	ATGTGCAGYACCAGTAARGTKATGGC<br>TGGGTRAARTARGTSACCAGAAYCAGCGG	*bla*CTX	593
1004<br>1005	GTGGTGGATGCCAGCATCC<br>GGTCGAGCCGGTCTTGTTGAA	*bla*CMY-1group	915
1006<br>1007	GCACTTAGCCACCTATACGGCAG<br>GCTTTTCAAGAATGCGCCAGG	*bla*CMY-2group	758
1062<br>1063	TCACTTTCATCACTGCGTTG<br>TTGGTCCATGACTACCAATG	*bla*OXA-*1*	820
1420<br>1421	ACGATAGTTGTGGCAGACGAAC<br>ATYCTGTTTGGCGTATCRATATTC	*bla*OXA-2	602
1359<br>1360	AGCCTCAGCAGCCGGTTAC<br>GAAGCCGTTAGTTGATCCGG	*bla*ACC-1	818

Running conditions were: 1 cycle of denaturation at 95 °C for 5 min, followed by 30 cycles of denaturation at 94°C for 60 sec, annealing for 60 sec at temperatures specific to each target— 54°C (*bla*TEM, *bla*CTX, *bla*CMY), 55°C (*bla*OXA), 60°C (*bla*SHV, and others), and elongation at 72 °C for 60 sec, and a final cycle of elongation at 72 °C for 10 min.

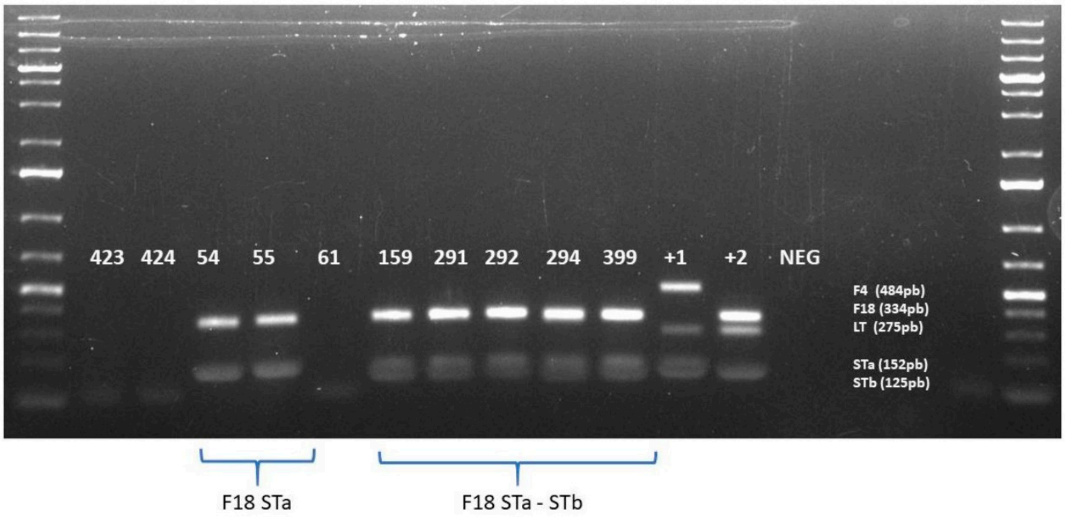

Figure S1. Multiplex PCR targeting fimbrial types (F4 and F18) and toxins (STxa, STxb, and LTx). Strains *E. coli* 423 – 399; +1 Positive control (F4, LT, Sta, STb); +2 (F18, LT, Stb); NEG Negative control, molecular weight marker (75–2000 bp).

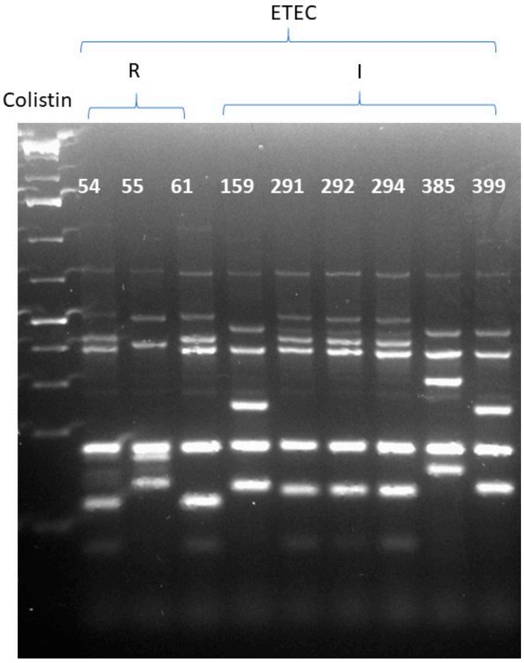

Figure S2. Multiple Locus Variable Analysis (MLVA) of ETEC strains. R: Colistin resistant MIC > 4; I: Colistin intermediate MIC < 2, molecular weight marker (75–2000 bp).

References

33. Do, K.H.; Byun, J.W.; Lee, W.K. Virulence genes and antimicrobial resistance of pathogenic Escherichia coli isolated from diarrheic weaned piglets in Korea. *J Anim Sci Technol* **2020**, 62, 543-552.
34. Zajacova, Z.S.; Konstantinova, L.; Alexa, P. Detection of virulence factors of Escherichia coli focused on prevalence of EAST1 toxin in stool of diarrheic and non-diarrheic piglets and presence of adhesion involving virulence factors in astA positive strains. *Vet Microbiol* **2012**, 154, 369–375.
31. Caméléna, F.; Birgy, A.; Smail, Y.; Courroux, C.; Mariani-Kurkdjian, P.; Le Hello, S.; Bonacorsi, S.; Bideta, P. Rapid and simple universal *Escherichia coli* genotyping method based on Multiple-Locus Variable-Number Tandem- repeat analysis using single-tube multiplex PCR and standard gel electrophoresis. *Appl Environ Microbiol* **2019**, 85, e02812-18.
37. Rebelo, A.R.; Bortolaia, V.; Kjeldgaard, J.S.; Pedersen, S.K.; Leekitcharoenphon, P.; Hansen, I.M.; Guerra, B.; Malorny, B.; Borowiak, M.; Hammerl, J.A.; Battisti, A.; Franco, A.; Alba, P.; Perrin-Guyomard, A.; Granier, S.A.; De Frutos Escobar, C.; Malhotra-Kumar, S.; Villa, L.; Carattoli, A.; Hendriksen, R.S. Multiplex PCR for detection of plasmid-mediated colistin resistance determinants, *mcr*-1, *mcr*-2, *mcr*-3, *mcr*-4 and *mcr*-5 for surveillance purposes. **2018**, 23, pii=17-00672.
38. Hasman, H.; Mevius, D.; Veldman, K.; Olesen, I.; Aarestrup, F.M. beta-Lactamases among extended-spectrum beta-lactamase (ESBL)-resistant Salmonella from poultry, poultry products and human patients in The Netherlands. *J Antimicrob Chemother* **2005**, 56, 115-121.
